# Literature Review of Long-Term Sequelae and Modern Management of Infantile Hemangioma

**DOI:** 10.7759/cureus.111442

**Published:** 2026-06-24

**Authors:** Kayla Coffman, Nicole Liang, Shane Cook

**Affiliations:** 1 Department of Dermatology, Marshall University Joan C. Edwards School of Medicine, Huntington, USA

**Keywords:** benign tumor of infancy, beta blocker therapy, cutaneous sequelae, infantile hemangioma, infantile hepatic hemangioma

## Abstract

Infantile hemangioma (IH) is a benign vascular tumor that occurs in neonates. Cutaneous IHs that are left untreated can result in permanent scarring and residual lesions. The presence of multiple cutaneous IHs can suggest visceral involvement and may require further workup. Management strategies for IH have evolved over the years. Originally, only life-threatening IHs were treated; however, recent literature has expanded our knowledge and supports several treatment options to reduce long-term sequelae. This review highlights recent literature on the epidemiology, pathogenesis, clinical presentation, differential diagnosis, complications, and treatment options for patients with IH. A PubMed literature search was performed using the keywords "infantile hemangioma", "residual lesion", and "sequelae", focusing on English-language articles published within the last 10 years.

## Introduction and background

Cutaneous infantile hemangioma (IH) is the most common benign vascular tumor in infancy [1]. IHs are caused by benign proliferation of vascular endothelial cells in the mesoderm [2]. IHs typically exhibit rapid growth in patients aged one to three months. Many IHs undergo involution; however, some exhibit incomplete regression, leading to permanent skin changes and creating functional and cosmetic complications. The location of the hemangioma is important in evaluating the risk of complications [3]. Clinical presentation and differential diagnosis of IH are important for early diagnosis and proper management. Complications of IH include long-term cutaneous sequelae and visceral involvement, making early initiation of treatment essential. Modern management of IH includes oral medications, topical medications, pulsed dye laser (PDL) therapy, and surgical options. This review serves to provide information so that medical providers can recognize IH, understand the long-term effects of untreated IH, and make clinical judgments regarding management.

## Review

Methods 

A literature search was conducted in PubMed for English-language articles using keywords including "infantile hemangioma", "residual lesion", and "sequelae". Eligible studies included original research, systematic reviews, meta-analyses, narrative review articles, and clinical practice guidelines relevant to IH. This narrative review was primarily limited to studies published within the last 10 years, with one earlier article to provide historical context regarding previous first-line treatment approaches. 

Epidemiology 

The presence of IH is most commonly noted one to two weeks after birth [2]. A meta-analysis published in 2024 included over 3,206 records and found the overall prevalence of IH to be 2.8%. IHs were more commonly found on the head and neck, with complications including ulceration in 16%, bleeding in 5.6%, visual impairment in 5.6%, infection in 2.8%, and subglottic obstruction in 1.5% [4]. Risk factors for the development of IH include female sex, low birth weight, preterm birth, infants born from multiple gestations, maternal progesterone therapy, and family history [5]. Infants with these risk factors should be monitored for the presence of IH, and caregivers should be educated about therapeutic options [4].

Pathogenesis

**Figure 1:**
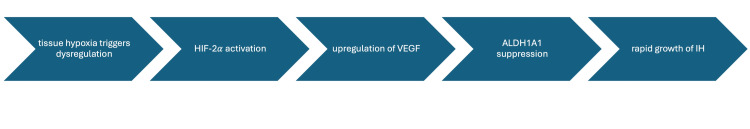
Proposed hypoxia-driven pathogenesis of infantile hemangioma HIF-2α: hypoxia-inducible factor 2 alpha; VEGF: vascular endothelial growth factor; ALDH1A1: aldehyde dehydrogenase 1 family member A1; IH: infantile hemangioma.

IH pathogenesis is complex and multifactorial, and many theories have been discussed in the literature. Dysregulated vasculogenesis and angiogenesis play a role in the development of IHs. Hypoxic conditions are described as a potential trigger for dysregulation (Figure 1). Newer studies suggest that the transcription factor hypoxia-inducible factor 2 alpha (HIF-2α) is important in active IH proliferation and increases as hypoxia persists. HIF-2α upregulates vascular endothelial growth factor (VEGF), leading to rapid growth. In later phases of IH, hypoxia signaling diminishes over time, leading to decreased HIF-2α and involution of the IH [6]. Additionally, under hypoxic conditions, mRNA expression of aldehyde dehydrogenase 1 family member A1 (ALDH1A1) is regulated. This enzyme is suppressed during the proliferation of IH, further suggesting that hypoxia is a trigger for IH development [7]. 

**Figure 2:**
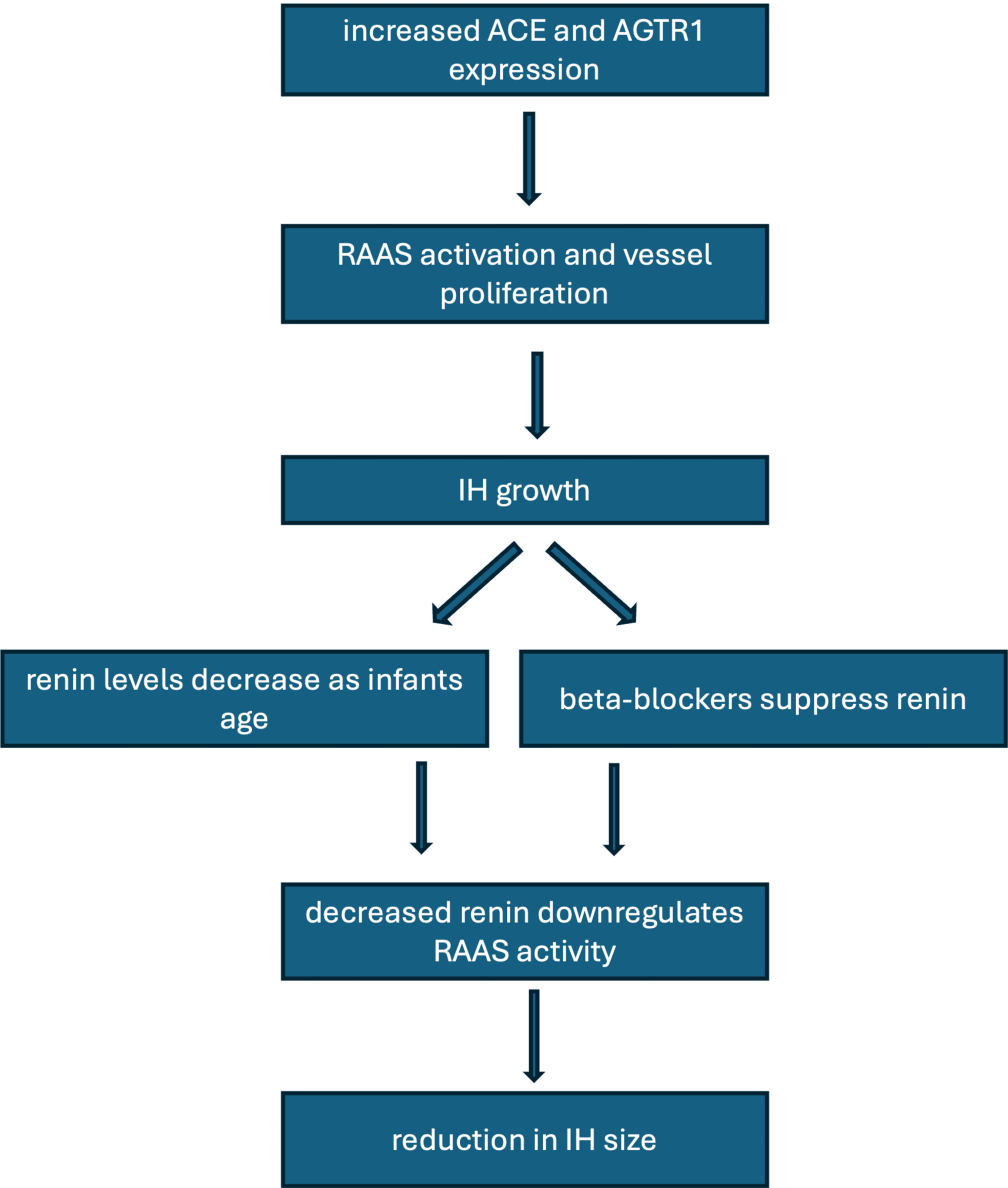
Proposed role of RAAS in infantile hemangioma pathogenesis ACE: angiotensin-converting enzyme; AGTR1: angiotensin II receptor type 1; RAAS: renin-angiotensin-aldosterone system; IH: infantile hemangioma.

Components of the renin-angiotensin-aldosterone system (RAAS) are upregulated during vessel proliferation (Figure 2). A study revealed elevated mRNA expression of angiotensin-converting enzyme (ACE) and angiotensin II receptor type 1 (AGTR1) in all stages of IH compared with the control group. The effects of the RAAS on IH could explain both the reduction in lesion size as patients age and the therapeutic effect of beta-blockers such as propranolol. Renin levels decrease over time as infants age, and beta-blockers suppress renin release, both contributing to downregulation of the RAAS [6]. 

The placental embolization theory suggests that IH stem cells originate from placental tissue that has migrated during fetal development and localized in the head or neck. GLUT-1 is strongly expressed in the placenta and IH endothelial cells; however, it is not expressed in other vascular tumors. This GLUT-1 expression provides support for this theory, along with the discovery of the potential biomarker chromosome 19 microRNA cluster (C19MC) [6]. C19MC is typically found only in the placenta; however, levels were elevated in patients with IH, correlated with tumor size, and decreased with propranolol treatment [8]. However, a study identifying aquaporin-1 (AQP1) in response to beta blockade in patients with IH concluded that placental endothelial cells express AQP1, whereas IH endothelial cells are AQP1-negative, suggesting evidence against the placental embolization theory [9].

Clinical presentation

The clinical presentation of IH depends on the distribution and depth of invasion. Precursor lesions are present at birth or arise soon after, appearing as focal areas of vasoconstriction or vascular erythematous macules. A latent period of one to three weeks is often observed before rapid tumor proliferation. Mature tumors can be classified into three broad categories by soft-tissue depth: superficial (strawberry), deep, and mixed. Superficial IHs are typically noncompressible papules, nodules, or plaques that penetrate the upper dermis. They appear bright red and often reach full size by three to five months [10]. Deep IHs develop in the deep dermis, often extending into the adipose tissue. They appear blue-tinted or skin-colored and are subcutaneous with irregular borders, but they may not become clinically apparent until one to three months of age. Growth can continue up to 12 months of age. Mixed IHs exhibit features of both superficial and deep IHs, involving the full thickness of the dermis with variable extension into the subcutaneous tissue. Clinically, they present with a bright red superficial layer and a blue-tinted deeper component. Patterns of distribution include focal, multifocal, segmental, and indeterminate. Focal IHs are localized to one area, and they tend to be smaller in size. Multifocal IHs present as discrete lesions at disparate sites, either as tiny lesions over the whole body or larger lesions in fewer locations. Segmental IHs are distributed geographically according to embryologic structures or the blood supply of embryologic arteries. Indeterminate distributions refer to lesions without a focal or segmental distribution [11].

Differential diagnoses

IHs appear similar to other vascular tumors, such as congenital hemangiomas (CH), and vascular malformations [11]. CHs arise from activating mutations in the GNAQ or GNA11 genes, leading to constitutively active MAPK. They tend to be more violaceous than IHs, and they can have prominent surface telangiectasias. They have usually achieved their maximum size at birth [12]. There are three subtypes of congenital hemangiomas: rapidly involuting (RICH), non-involuting (NICH), and partially involuting (PICH). RICHs typically fully involute by 8-14 months of life, while NICHs persist, growing proportionally with the child. PICHs decrease in size but never completely disappear [13]. A detailed history and physical examination may be able to differentiate IHs and CHs, but the distinction often is not clear. GLUT-1 immunohistochemistry on a biopsy specimen can provide a definitive diagnosis. IHs are always GLUT-1 positive, while CHs are GLUT-1 negative [10]. It is important to consider other vascular tumors, such as pyogenic granulomas, Spitz nevi, and Kaposiform hemangioendothelioma. Vascular malformations, such as port-wine stains, lymphatic malformations, and arteriovenous malformations, can also present similarly. These can typically be distinguished via dermoscopy or high-resolution ultrasound. They are also negative for GLUT-1 [11]. A diagnosis of IH should be confirmed before any treatment is attempted [10].

Complications 

Cutaneous Complications

A wide range of sequelae can occur after involution of cutaneous IH, including telangiectasia, fibrofatty tissue, redundant skin, and scarring. A systematic review showed that sequelae rates for small superficial and well-treated IHs were around 5%-10%, whereas large untreated IHs can leave residual skin changes in greater than 90% of patients. These recent studies show that treatment does not eliminate the risk of residual skin changes after the involution of IH but certainly decreases the risk and severity [1]. Recent studies show that single or combination therapy with pharmacologic and/or laser treatments reduces residual lesions in patients. Among untreated IH patients, 75.2% had at least one residual lesion compared with 41.4% of patients treated with single or combination therapy [14].

High-Risk Anatomical Site Complications

Lesions near the eye can result in vision disturbance, while lesions near the mouth can lead to feeding difficulties. There is a high risk of disfigurement in places such as the tip of the nose and ears, along with an increased chance of scarring in patients with lower facial hemangioma. The risk of disfigurement in patients with IH is much greater than the risk of life-threatening complications. Between the 1950s and 1980s, experts recommended treatment only in cases of functional impairment. Modern studies have revealed that permanent skin changes in the referral setting can be seen in 55% to 69% of those with untreated IH. These findings have resulted in support for treatment initiation to reduce cosmetic deformity and improve future quality of life [3].

Visceral IH Involvement

Five or more cutaneous IHs should lead to abdominal ultrasonography to rule out infantile hepatic hemangiomas (IHH) [3]. IHH can lead to life-threatening complications such as consumptive hypothyroidism, congestive heart failure, abdominal compartment syndrome, thrombocytopenia, and coagulopathy. Patients with hepatic involvement should be referred to pediatric endocrinology for further workup regarding hypothyroidism. IHH is caused by overexpression of type 3 iodothyronine deiodinase in the hemangiomas, resulting in a different presentation compared to congenital hypothyroidism. Untreated hypothyroidism in these patients can lead to devastating consequences, including severe neurological damage and low-cardiac-output failure. Patients presenting with abdominal distention and hepatomegaly should be urgently evaluated for abdominal compartment syndrome, even in patients with few to no cutaneous hemangiomas. IHH will most likely proliferate within the first few weeks of life, making screening in the first few days after birth unhelpful. Abdominal ultrasounds can be performed in the first or second month of life in patients with five or more cutaneous hemangiomas or if there is any suspicion of visceral involvement, regardless of cutaneous involvement. Although the majority of IHHs will involute, a portion of these patients with diffuse hemangiomas have a high risk of mortality. It is important for health care providers to use clinical judgment regarding initiation of treatment, and medical therapies can be initiated to hasten the resolution of the hemangiomas. Pharmacologic options for IHH include corticosteroids with propranolol or vincristine. If pharmacologic therapies do not achieve an appropriate response, embolization can be used. Liver transplantation is not commonly needed but can be a last-line therapy for patients with diffuse presentations in whom medical and embolization therapies have offered no benefit [15].

Associated Syndromes

Large segmental cutaneous infantile hemangiomas (IHs) greater than 5 cm should warrant investigation for PHACES (posterior fossa malformations, hemangioma, arterial anomalies, cardiac defects/coarctation of the aorta, eye abnormalities, and sternal defects) or LUMBAR (lower body hemangioma, urogenital anomalies/ulceration, myelopathy, bony deformities, anorectal malformations/arterial anomalies, and renal anomalies) syndrome, which includes a variety of congenital abnormalities. Over 90% of patients with PHACES syndrome have cerebrovascular anomalies. The most common extracutaneous finding in LUMBAR syndrome is spinal dysraphism [3,11].

Treatments

The most widely used modern pharmacologic options for IH include both oral and topical nonselective beta-blockers. The use of these medications tends to shorten the natural course of the lesion and suppress lesion proliferation. Nonpharmacologic options include pulse dye laser (PDL) therapy and surgical intervention [2].

Corticosteroids

In modern management, beta-blockers remain the first-line treatment for IH. Prior to the use of beta-blockers, corticosteroids were commonly used because of their anti-inflammatory effects. Both oral corticosteroids and intralesional steroids were utilized. Over the years, studies have shown that beta-blockers are more effective and have fewer adverse effects compared to corticosteroids. Corticosteroid side effects include weight gain, hyperglycemia, immune suppression, behavior changes, hypertension, and growth suppression [16]. A meta-analysis from 1965 to 2012 revealed that, out of 1,162 studies, patients treated with corticosteroids for one to three months had a pooled response rate of 69%, while propranolol-treated patients treated for 1-12 months had a 97% response rate. Steroid-treated patients had an adverse effect rate of 17.6% compared to 9.6% in propranolol-treated patients [17]. Beta-blocker therapies can be used for a longer duration and do not possess the adverse effects seen with corticosteroids after two weeks of use. The use of corticosteroids may still be considered in cases where beta-blocker therapy is contraindicated or ineffective [16].

Oral Beta-Blockers

Oral propranolol is the first-line pharmacological approach for IH causing functional impairment [2]. A recent prospective trial evaluated outcomes of oral propranolol treatment at 1-3 mg/kg/day with response-based titration for facial IH over four years. A total of 272 patients were involved in the study, with 14.0% diagnosed with ulcerated IH. Following oral propranolol treatment, 97.4% of patients had a significant response to therapy, and 85.3% had no or minimal sequelae. Segmental, mixed, and lip IHs were independent risk factors for major rebound, and 16.9% had rebound after discontinuing propranolol therapy. Surgical interventions were needed in 8.1% of patients after therapy, while 8.8% of patients required laser treatment [18].

Oral propranolol is a first-line option for PHACES and LUMBAR syndrome because of its ability to cross the blood-brain barrier. A European multicenter observational retrospective study published in 2020 used oral propranolol in seven patients aged three to seven months. Treatment was discontinued when patients had complete or almost complete resolution of cutaneous and CNS hemangiomas after an average of 6-14 months of treatment. Propranolol can still be beneficial in cases requiring surgical intervention to decrease hemangioma size and the risk of bleeding [19].

Atenolol is a selective B1-blocker that may offer fewer adverse effects, such as bronchospasm and CNS effects, compared to nonselective beta-blockers. In a clinical analysis, 133 patients with proliferating IH were treated with oral atenolol for a mean duration of approximately five months. The data revealed no true difference in response rate between atenolol and propranolol; however, atenolol may have the benefit of lower doses and fewer adverse events. More evidence is needed to support the use of atenolol as first-line therapy, but it may be considered in patients who do not tolerate propranolol [20].

Topical Beta-Blockers

Topical propranolol is particularly helpful in patients with small superficial hemangiomas that do not require systemic treatment. Topical treatments also avoid the systemic side effects of nonselective beta-blockers, such as bradycardia and hypoglycemia. A systematic review revealed that topical propranolol led to improvement of lesions in 90% of patients, with a 50% reduction in lesion size in 59% of cases. Topical propranolol concentrations utilized ranged from 0.5% to 5%, with treatment durations ranging from 2 weeks to 16.5 months. The review concluded that beginning treatment before three months of age was associated with better outcomes compared to later initiation of treatment [21].

Topical timolol was found to be a safe option for the treatment of localized small IHs and was found to be superior to watchful waiting for superficial IHs in high-risk areas [2]. A retrospective analysis included over 600 patients with IH treated with topical timolol between 2019 and 2023. The study concluded that patients between one and three months of age with IHs between 1.5 cm and 5 cm had better outcomes. Residual skin lesions occurred in 3.9% of cases, and 8.8% of cases relapsed [22].

Nonpharmacologic Therapies

PDL is another treatment used for IH; however, it is best utilized in addition to a nonselective beta-blocker. A double-blind study of 30 infants compared the use of PDL alone with PDL plus timolol gel 0.05%. The results supported that the combination treatment achieved greater lesion size reduction with cost benefits. The use of timolol with PDL was preferred because of its high efficacy and time efficiency compared to PDL alone [3]. Laser therapy is also an option for treating skin changes and scarring following the resolution of IH [16].

Surgical interventions are rarely indicated in infancy, and the highly vascular nature of these tumors makes resection less favorable. Surgery is needed in cases where vital structures may be compromised and pharmacological therapies have failed. Surgery can also be considered in situations where early intervention will allow a better reconstructive result in the future. The majority of IHs will not improve after three to four years of age; therefore, if surgery is indicated for residual skin changes, this age range is preferred rather than waiting until the child is older. Tumors in this age range are less vascular and reduced in size, resulting in a better surgical outcome and smaller scars [3].

Emerging Therapies

Additional therapeutic options for IH are under investigation. Targeted propranolol formulations are a potential future treatment option and are currently in the preclinical stage. Targeted drug therapy has the potential benefits of increased drug concentration at the target site, low toxicity, the ability to cross biological barriers, and controlled in vivo release. There are a variety of drug carriers, including liposomes, micelles, microspheres, and others. These targeted therapies aim to reduce the side effects seen with oral propranolol preparations and be more efficacious than topical preparations currently on the market. The application methods of targeted agents are variable and include transdermal delivery, intratumoral injections, intraperitoneal injections, and others. Although toxicity rates, efficacy in clinical trials, and drug delivery systems must continue to be studied, these therapies have the potential to offer alternative treatment options to patients in the future [23].

## Conclusions

IHs can be classified as superficial, deep, or mixed. Differential diagnoses must be considered, including congenital hemangiomas and vascular malformations such as port-wine stains, lymphatic malformations, and arteriovenous malformations. The clinical sequelae range from persistent cutaneous alterations to severe life-threatening complications. The psychological impact of residual skin changes represents an important area for future research and may provide further insight into the overall quality-of-life burden associated with this condition. Corticosteroids and observation were the mainstays of treatment before the introduction of beta-blocker therapies. Beta-blockers, including oral propranolol, have become the first-line treatment option over the past several years. The utilization of laser therapy and surgical intervention is limited to certain circumstances and should be considered in select cases. Emerging therapies include targeted drug delivery systems that have the potential to increase drug efficacy and decrease toxicity.

This literature review highlights the ongoing need for early identification and appropriate treatment of IH. Initiation of treatment should take into account the long-term risks and benefits of pharmacologic or laser therapy. Further research into IH treatments will result in more efficacious treatment options with fewer adverse effects. Continuing clinician education in the primary care setting and during early medical education will help raise awareness and improve outcomes for patients with IH.
